# Supplementary material for: Are carbon emissions trading and green financial instruments synergistic? -Comprehensive quantitative research based on content analysis
Source: PLoS One. 2024 Mar 7;19(3):e0298601. doi: 10.1371/journal.pone.0298601 (PMC10919601; doi:10.1371/journal.pone.0298601)
Supplement: S1 Data — (ZIP) [file pone.0298601.s001.zip › Data/地级市Z_F工作报告.zip!/┴╔─■/░░╔╜╩╨/└┤╫╘╣1⁄2╓┌║┼í╛┬φ┐╦╩2╛▌═°í┐_2014.docx]

各位代表：

现在，我代表市第十五届人民政府向大会报告工作，请予审议，并请市政协各位委员提出意见。

一、2013年政府工作回顾

2013年，面对严峻复杂的经济形势，市政府在省委、省政府和市委正确领导下，在市人大、市政协监督支持下，紧紧依靠全市人民，牢牢把握“稳中求进”总基调，攻坚克难，真抓实干，较好完成了市十五届人大一次会议确定的各项工作任务。预计全年实现地区生产总值2638亿元，增长9%；公共财政预算收入239.2亿元，增长2.1%；固定资产投资1970亿元，增长20%；社会消费品零售总额800.8亿元，增长13.8%；城镇居民人均可支配收入27097元，增长12%；农民人均纯收入14257元，增长13%；万元地区生产总值综合能耗下降3.8%。主要经济指标增幅高于全省平均水平。

（一）全力以赴抓经济运行，稳增长成效明显

坚持把稳增长作为重中之重，积极应对经济下行压力，各项经济指标呈现逐月向好的回升态势。

围绕企业抓运行初见成效。把企业作为稳增长的核心，通过召开中省直企业和重点民营企业家座谈会，落实市政府领导包保重点企业责任制，组织银企对接会，千方百计帮助企业解决历史遗留问题和生产经营困难。设立3亿元工业企业转贷资金和1亿元钢铁企业开拓市场专项资金，为130户企业补办土地证和房产证，帮助企业融资30多亿元。全年新增规模以上工业企业120户。完成规模以上工业增加值1070亿元，增长10.5%。

招商引资上项目成果显著。以项目建设年为主线，持续不断赴香港、上海、浙江、江苏、广东、福建等地招商，全力推进项目落地和开工建设。全年开工建设亿元以上项目760个。实际利用外资13.8亿美元，增长8.5%；引进域外资金1248亿元，增长20%；外贸出口27亿美元，增长13.5%。金融机构新增本外币贷款170亿元。

地企合作不断深化。全面加强与鞍钢战略合作，积极为鞍钢扭亏增效、结构调整和发展非钢产业服务。鞍钢生产经营平稳回升，全年完成增加值210亿元，增长4%，实现扭亏增效目标。鞍钢等中省直企业为稳增长做出了积极贡献。

科技创新能力不断增强。召开全市科技创新大会，出台《加快推进科技创新实施意见》，以企业为主体的技术创新体系建设成效显著。积极推进企业和清华大学、中科院自动化所等高校院所开展产学研合作和技术转移项目537项。围绕主导产业设立院士工作站6个。东北大学鞍山激光应用技术研究院、大连理工大学鞍山研究院签约落户。新增国家高新技术企业23家。获评省创新型中小企业54家。完成高新技术产品增加值437.6亿元，增长30.6%。全社会研发投入占GDP比重1.69%,比上年提高0.13个百分点。再次荣获全国科技进步先进市，成为五连冠城市。

（二）坚持不懈抓转型升级，调结构步伐加快

坚持以工业集群化、服务业集聚化、农业产业化为重点加速转型升级，三次产业比重达到4.2:54.4:41.4。

新兴产业集群突飞猛进。在做深做优钢铁深加工、装备制造、菱镁新材料、轻纺等传统产业的同时，大力发展激光科技、高端阀门、彩涂板、精细化工、新能源电池、健康制造等新兴产业集群。激光产业创造“激光速度”，新签约入驻东莞盛雄等103户企业，获批国家火炬特色产业基地，成功举办全国激光产业技术交流会和沈阳经济区新兴产业集群现场会，成为全省新兴产业一面旗帜。高端阀门产业增势强劲，成功召开首届中国（鞍山）高端阀门产业博览会，投资15亿元的丹佛斯全球供热设备制造基地部分建成，日本工装等世界500强企业相继入驻，全年投资增长8.7倍。彩涂板产业异军突起，辽宁·台安彩涂板产业招商推介会成效显著，辽宁银恒等7个项目竣工投产，河北华德等16个项目开工建设,全省县域工业产业集群现场会在台安召开，成为全省县域产业集群发展样板。煤焦油及粗苯深加工产业基地与鞍钢签署框架协议，中国国际商会汤岗子健康产业基地部分项目开工建设。全年新兴产业投资增长49.4%，高出传统产业24个百分点。

服务业集聚区加快建设。铁东中央商务商贸、汤岗子新城旅游休闲、达道湾现代服务业、海城西柳专业市场等14个服务业集聚区加速发展，省级服务业集聚区数量达到7个。投资150亿元的港中旅温泉城、投资100亿元的港龙国际城市综合体等195个项目开工建设，宝马钢铁物流中心等10个重点项目竣工营业。发展假日经济、夜经济效果明显。新引进营口银行、锦州银行、安华保险、信诚寿险等8家金融机构，鞍山农村商业银行股份公司正式营业。成功举办首届玉佛山庙会等大型活动，接待国内外游客3870万人次，旅游总收入增长33%。成功举办春夏秋房交会，房地产投资额增长18.5%，商品房销售额增长15%。全年完成第三产业增加值1092亿元，增长6.8%。

农业产业化蓬勃发展。全年粮食产量148.8万吨，创历史最高水平。全市机插秧率89%，基本实现水稻全程机械化。海城南果梨产量18万吨，优质大蒜产量5万吨；台安鸭鹅饲养量达到2亿只，获批全省“一县一业示范县”；岫岩食用菌产量恢复到60万吨，保持全国“蘑菇第一县”称号。全市有9个十亿元以上农产品深加工项目开工建设，海台岫农高区新入驻福建安井集团速冻食品等18个亿元以上重点项目。全市县域公共财政预算收入增长18.7%，位列全省第三。

（三）坚定不移抓体制机制创新，促改革效果显著

统筹推进简政、放权、扩容、增活“四位一体”行政体制改革，全面释放城区、开发区经济发展活力。

完成新一轮区划调整。将文化旅游开发区、农业高新技术产业园区成建制划入汤岗子新城管理，将千山镇划入高新区管理，将大孤山街道划入铁东区管理，进一步优化了城市空间布局，拓展了城区、开发区发展空间。

向城区开发区放权力度加大。出台《向城区和开发区下放市级管理权限的意见》，在省要求下放的22项权限基础上，向城区下放52项市级管理权限，向高新区、经济开发区、汤岗子新城下放54项市级管理权限，城区、开发区发展活力全面增强。全市城区公共财政预算收入增长17.1%，增速全省第一。

开发园区活力有效释放。出台《推进开发园区体制机制创新实施意见》，按照“减少层级、精简机构、理顺关系、提高效能”原则，对全市21个开发园区实行党政合一体制和“扁平化”管理模式，面向全社会招聘选拔优秀人才。全年开发园区完成固定资产投资954.1亿元，增长24.5%，成为全市经济发展的主战场。

简化行政审批取得突破。出台《鞍山市建设工程项目优化审批流程方案》，全面清理和精简建设项目审批事项，将项目立项到施工许可的审批时间由203天压缩到最短32天。调整4个单位的行政管理体制，政府组织结构进一步优化。

（四）统筹兼顾抓城乡建设，人居环境明显改善

坚持把完善基础设施和改善人居环境作为重点惠民工程来抓，城市承载能力和城乡环境质量显著提高。

城乡建设管理不断加强。总长55.5公里的兴业大道主体竣工。四达大道、奥育街等48条道路新建改造工程和千山西路、五一路等183条道路维修养护工程全面完成。建国大道、鞍山路等重点道路加快建设。建成长途客运北站。腾鳌机场盲降系统建成使用，开通鞍山至北京、上海航线。高标准完成千山东路、兴业大道等绿化工程199万平方米，全市绿化、美化、亮化、净化水平显著提升。开展整治非法营运、超载超限、占道经营、行人闯红灯、城市犬害等专项行动，有效提高城市管理水平。全面实施城乡生活垃圾清运处理专项行动，集中整治城乡结合部、集贸市场等卫生死角，营造优美整洁的城乡环境。

新城新市镇加速建设。汤岗子新城建设取得重大进展，新城“五横六纵”主干路网、中央活力区分支路网和凤凰湖核心景区建设全速推进，台湾京辉京华城、香港南宇七星会馆、东泰盛唐国际五星级酒店等11个重点项目全面开工，汤岗子新城中心医院、鞍山师范学院新校区、新加坡天丞国际学校等8个社会事业项目全面启动。达道湾、腾鳌、海西、析木、八角台、玉都新城和牛庄新市镇建设全面推进。海城、台安、岫岩加快推进基础设施、公共设施、行政中心和商务中心建设，完成县域房地产开发面积878万平方米。

生态环境建设全面加强。出台《蓝天工程实施方案》，召开省蓝天工程现场会，与鞍钢等重点企业签署治理责任状，安排总投资188亿元，全年开工87个重点项目，鞍钢二烧厂烧结机脱硫、冀东水泥回转窑脱硝等46个项目竣工。青山工程完成小开荒还林3.49万亩，退坡地造林4.91万亩，封育围栏1103公里，矿山生态环境治理1.1万亩，墓园绿化12.2万株，青山工程综合排名全省第一。碧水工程完成达道湾等4座污水处理厂提标改造和东台等4座污水处理厂管网配套，城镇污水处理率达到85%。污染30余年的运粮河得到有效治理，彻底改善了周边8个村3万多名群众生活环境，提升了高铁西站城市窗口形象。

（五）全心全意抓民生改善，惠民生取得实效

以为城乡群众办20件实事为重点，努力让发展成果惠及广大人民群众。

民生工程积极推进。高度重视回迁房建设，投资33.5亿元，新建续建33个回迁房项目，建成回迁房86万平方米，安置居民4814户。全年城镇实名制就业11.5万人，城镇登记失业率下降到1.97%。开展企业养老保险扩面征缴百日行动，全市企业退休职工养老金人均增长166元。在全省率先启动22种重大疾病保障工作，新农合住院报销比例提高到75%。部分优抚对象抚恤补助标准提高15%。城市和农村低保年标准分别提高720元和480元。物价保持总体稳定。新建改造100个示范性养老服务中心和37所农村敬老院。资助5万名困难家庭学生就学。为1万名计划怀孕群众实施免费健康检查。引进200辆清洁能源公交车。新建维修改造供暖锅炉92台、管网102公里。恢复重建320公里水毁农村公路。加大信访问题排查化解力度，社会保持和谐稳定。全年未发生重特大生产安全事故和食品安全事件。推进社会治安防控体系建设，严厉打击各类违法犯罪，人民群众安全感和满意度进一步增强。

社会事业全面发展。完成30所城乡公办幼儿园和145所标准化中小学建设改造任务，铁东、立山进入省基础教育强县区行列。市职教城直属5所中职学校分别进入国家和省级示范校行列。新建50个社区图书馆，图书馆“一卡通”实现全地区图书资源共享。新建成电影大厦。完成有线电视数字化整转工作。全面推行市属公立医院药品集中采购供应管理。引入社会资本参与第二医院等公立医院改革。完成肿瘤医院整体建设。试行儿童白血病、先天性心脏病和肾病透析患者免费治疗工程。奥体中心一场三馆建成使用，圆满完成十二运赛事组织安保工作。新建改造100个城乡健身广场。人口与计划生育、妇女儿童、老龄、民族、宗教、外事、侨务、对台、残联、统计、接待、史志、档案、气象、地震、人防、民兵预备役、双拥共建等工作都有新成绩。

（六）求真务实抓政府自身建设，行政能力不断提高

把求真务实作为政府工作的自觉追求，着力建设行为规范、运转协调、公正透明、廉洁高效政府。

民主法制不断加强。自觉接受市人大及其常委会法律监督、工作监督和市政协民主监督。全年办理人大代表建议、批评和意见189件，办理政协提案426件。提请审议地方性法规草案2件，制定和修正政府规章5件，受理行政复议案件37件。推行政府信息公开，保证广大群众知情权、参与权、监督权。

行政效能显著提升。严格执行目标管理绩效考核、行政效能监察和责任追究制度，对各项工作任务实行旬分析、月调度、季通报，做到一级抓一级，层层抓落实。启动网上审批运行平台、重大项目服务直通车和外商投资企业联合年检绿色通道，优化投资发展软环境。加强公共资源交易中心建设，实现公共资源“阳光”交易。

工作作风更加扎实。学习贯彻习总书记系列讲话精神，积极倡导勇于担当、敢啃硬骨头的工作作风，坚持做打基础利长远的实事和解民忧惠民生的好事，有效破解了一系列历史遗留问题。解决了25宗已出售土地腾净地问题，努力化解了银座、国际酒店、化纺厂等国企改制难题，严格控制债务规模、债务成本，着力防控债务风险。各部门勇于放弃部门利益，推动行政体制改革逐步走向深入。领导干部带头执行廉洁自律各项规定，树立了为民务实清廉的良好形象。

各位代表，过去一年，我们用坚韧和勇气顶住了经济下行的巨大压力，用勤劳和智慧逐步破解了发展的瓶颈难题，用拼搏和汗水赢得了来之不易的发展成果。这些成绩的取得是在市委正确领导和市人大、市政协鼎力支持下，全市各县（市）区、各部门团结一心、克难攻坚的结果，是广大干部群众解放思想、大胆创新的结果，是鞍钢等所有企业忘我拼搏、战胜困难的结果，更是全市上下政通人和、凝心聚力的结果。在此，我代表市政府向为鞍山发展付出辛劳的各位代表和委员，向奋战在全市各条战线的广大干部和群众，向各民主党派、工商联、无党派人士、各人民团体，向中省直单位、驻鞍部队、武警官兵，向关心支持鞍山发展的老领导、老同志，向所有为鞍山发展做出贡献的海内外朋友，表示衷心的感谢和崇高的敬意！

各位代表，在总结成绩的同时，我们也清醒地看到鞍山经济社会发展还存在许多矛盾和问题。主要是工业新兴产业比重低，大企业大项目少；服务业增速较慢；鞍海一体化进展不快；生态环境建设和节能减排任务艰巨；市场化程度不高，政府行政效能和软环境建设还需要进一步加强；在回迁房建设、盘活存量资产、城市规划和城市管理等方面群众反映强烈。这些问题，我们将在今后工作中牢记于心，努力破解。

二、2014年主要工作任务

2014年是全面深化改革的开局之年，也是全面实现“十二五”规划目标的重要一年。新一年政府工作的总体思路是：全面贯彻落实党的十八大、十八届三中全会和中央、省、市经济工作会议精神，牢牢把握“稳中求进”总基调，以改革创新为统领，加速结构调整和转型升级，加快推进新型城镇化，加强生态环境和基本公共服务体系建设，着力改善民生，促进社会和谐稳定，积极创建全国文明城市，全面建设实力鞍山、诚信鞍山、素质鞍山、幸福鞍山、美丽鞍山。

2014年主要预期目标是：地区生产总值增长9%，公共财政预算收入增长9.5%，固定资产投资增长20%，社会消费品零售总额增长10%，城镇居民人均可支配收入增长9%，农民人均纯收入增长10%。

（一）全力推进改革开放

按照十八届三中全会要求和市委的总体部署，以更大的决心、更务实的作风和更勇于创新的精神，全面深化各领域改革。

深入推进向城区开发区放权。按照“放得下、接得住、管得好”原则，对已下放权限落实承接情况进行检查指导。市直有关部门做好下放权限的对接和监管，做到扶上马、送一程。各城区、开发区做到机构到位、人员到位、培训到位、承接到位。进一步解放思想，将由城区、开发区管理更方便有效的经济社会权限继续下放，做到人随事走，机构随人走。按照“大部制”原则，优化机构职能，严控人员编制，提高行政效能。

继续推进园区体制机制创新。加大对各开发园区的督促检查力度，对招商引资、项目进展、财政收入等实行量化考核，细化考核指标，强化目标管理和责任落实。推进行政审批、财税统计、选人用人和收入分配等全方位改革落实到位。使各开发园区成为全市经济发展的主战场和体制机制创新的活力区。

大力推进向市场放权。充分发挥市场在资源配置中的决定性作用。政府主要加强发展战略、规划、政策、标准制定和实施，加强市场监管和公共服务。按照“非禁即入”原则，凡负面清单之外领域，一律允许各类企业依法平等进入。做到该管的管住，能放的都放。加强要素市场建设，深化国有企业股权多元化改革。加强公共资源交易中心规范化建设，提高公共资源交易质量和效率。发挥公共行政服务中心载体作用，进一步优化行政审批流程，大幅度实质性减少行政审批事项。除国家规定的项目外，一律由企业自主决策、自主投资、自主经营。

积极推进向社会放权。大力培育律师、会计、咨询、评估等中介机构和商会、协会等社会组织。加快事业单位分类改革，推动公办事业单位去行政化，推进有条件的事业单位转为企业或社会组织。加大政府购买服务力度，凡属事务性管理服务，原则上都要引入竞争机制，向社会购买，鼓励社会力量参与基础设施建设和公共服务。

全面扩大对外开放。培养和组建一批熟悉产业发展、满怀工作激情的招商队伍。面向港台、珠三角、长三角等重点地区，围绕行业龙头企业、上市企业、待上市企业、鞍钢上下游及配套企业，开展专业化招商、点对点招商、代理招商、以商招商，积极引进符合鞍山当前及长远发展的重大项目。重点抓好已洽谈项目的跟踪落实。加强外贸出口基地建设，优化出口产品结构。积极盘活存量资产，防控债务风险，拓宽融资渠道。全年开工建设亿元以上项目800个。实际利用外资增长8%，引进域外资金增长20%，外贸出口增长9%。

发挥科技创新引领作用。强化企业技术创新主体地位，建立主要由市场决定的科技创新体制机制。发挥政府引导和激励作用，构建技术研发、人才培养、检验检测等专业化服务平台。支持创新型龙头企业，培育创新型中小企业，鼓励高端科技人才创新创业，建立基于全产业链的技术创新战略联盟。全年新增国家高新技术企业20家，高新技术产品增加值增长20%。

（二）全力打造工业强市

坚持工业强市不动摇，深入实施“工业五项工程”。全年新增规模以上工业企业100家，规模以上工业增加值增长10%。

迅速做大新兴产业集群。激光科技重点扩大高功率激光器应用领域，构建激光通信、激光加工、激光医疗、光机电一体化全产业链，全年实现产值100亿元，5年达到1000亿元，打造国家级激光产业基地。高端阀门重点加快东北最大阀门检测中心和30万平方米工业地产建设，集聚优秀阀门企业，打造国内最大高端阀门产业基地。彩涂板重点引进企业总部，加快进口国外关键设备，拓展家居家电行业板材产品，出口欧美市场，打造东北最大彩涂板生产研发基地。煤焦油深加工全面落实与鞍钢战略框架协议，加快规划布局和基础设施建设，引进一批深加工项目，打造国家级煤焦油深加工产业基地。健康制造依托中国国际商会汤岗子国际健康产业基地，大力引进食品、药品、保健品、化妆品、高端运动和医疗器械重点项目，打造千亿产值健康制造产业集群。依托临港优势，发展大洋河高分子材料产业。围绕节能环保设备制造和资源循环利用，发展节能环保产业。全年大力推进雷盛电子激光电源研发制造基地、河北神华100万吨镀锌钢带、丹佛斯全球供热设备制造基地、60万吨煤焦油深加工、上海复星医药、汉能科技300MW第三代薄膜太阳能电池制造基地、华塑高分子新材料产业园等220个重点项目建设。

改造提升传统产业集群。钢铁深加工围绕特殊钢、精品钢材、板管线型材深加工和钢构七大板块，提升工艺设备水平，提高产业附加值，打造世界级钢铁深加工产业基地。装备制造围绕冶金矿山装备、电力电控装备、工程机械、汽车零部件和通用机械等六大板块，提高研发、设计和制造能力，推进“鞍山制造”升级为“鞍山创造”。菱镁新材料坚持资源开发和节约并举，围绕镁耐材、镁合金、镁建材、镁制药和镁化工五大板块，打造世界级菱镁新材料产业基地。纺织服装加速形成纺织、印染、服装设计加工完整产业链，打造国内领先的纺织服装基地。全年大力推进鞍山紫竹重型铸件标准化厂房、鞍山宏源多功能排水器、辽宁华宇玻镁板生产线、海城鑫泰衬布生产线等880个重点项目建设。

大力支持鞍钢等中省直企业发展。支持鞍钢投资10亿元的180吨转炉异地改造、投资2.8亿美元的高强度汽车板等项目加速建设，推进煤焦油、苯加氢等项目扩大产能。支持鞍钢发展金融贸易、工程技术、化工、信息技术、物流能源、房地产等非钢产业。推进本地企业与鞍钢合作，发展产业配套和外包服务。支持中冶集团、中钢集团、中船重工等驻鞍企业做大做强。

大力扶持民营企业发展。全面落实支持非公有制经济和中小微企业发展政策措施，激发非公有制经济创造力。继续实施市政府领导包保重点企业责任制，在资金、技术、人才、市场和项目建设等方面，主动帮助企业解决实际问题。支持各类企业之间开展合资合作、项目融资、品牌建设、并购重组，在企业产权制度创新上实现突破。

（三）全力推进服务业提档升级

以国家现代服务业发展试点市为契机，推进服务业发展取得新突破。全年开工建设亿元以上服务业项目130个，第三产业增加值增长8%。

优先发展生产性服务业。围绕主导产业集群，大力发展总部经济、楼宇经济和工业企业分离生产性服务业。培育发展风险投资基金、种子基金和天使基金；支持高新区发展科技金融，培育发展科技银行、科技小贷公司；进一步引进优质金融机构，丰富金融业态；支持鞍山银行实现跨区域经营。加快建设西柳服装城、南台箱包城、新新水暖城、义乌小商品城、东四家俬城等西柳至南台40平方公里专业市场集群，打造集生产加工、市场销售、仓储物流配送为一体的东北专业市场集聚区。扶持东北矿产品交易中心、东北钢铁网、中国煤焦化商务网、恒瑞贵金属网、海德镁制品供应链等生产资料电子商务平台，办好中国西柳国际服装节、辽宁南台箱包博览会、能源装备制造产业博览会等专业品牌展会。推进激光、阀门、菱镁检测研发等公共服务平台建设，积极发展研发设计、文化创意、信息服务产业。

升级发展消费性服务业。大力推进汤岗子新城北京慈铭中心、美国双兰基地、中青旅基地、国际重阳节永久会址等重点项目建设，发挥汤岗子疗养院品牌优势，把健康管理和养老产业作为拉动全民消费、促进现代服务业发展的重大举措，不断拓展新兴消费领域。围绕铁东中央商务区扩容，加快推进红星国际广场、万熹城市广场、银光购物中心等重点项目建设，发展集购物、餐饮、休闲、娱乐为一体的体验式消费。发展假日经济、夜经济，拓展居民消费市场。

大力发展旅游产业。加快千山风景区核心景区保护性建设和千山国际文化旅游产业园等重点项目建设，争创国家5A级景区。加快玉佛山市民休闲及文化旅游设施建设，创出玉佛山庙会品牌。加快发展温泉旅游、沟域旅游、乡村旅游、文化旅游、商贸旅游，提升知名度和美誉度，把鞍山建成中国北方重要旅游目的地城市。全年接待国内外游客和旅游总收入双增长25%以上。

推进房地产业健康发展。支持房地产开发和建筑企业发展壮大，积极引进知名房地产开发企业，提升商品房品位和档次。全面开工回迁房建设，完成7个项目94万平方米回迁房任务，让动迁居民早日回迁。加快老城区、棚户区、城中村改造，积极向上争取7万套棚户区、城中村改造政策，推进货币化安置，加快保障性住房建设步伐。全年实现房地产投资额和商品房销售额双增长10%以上。

（四）全力推进农业现代化

用工业理念谋划农业，推进农业现代化取得新突破。全年县域地区生产总值增长11%，公共财政预算收入增长12%，固定资产投资增长25%。

加速推进“一县一业”。海城重点推进南果梨提质增效和大蒜机械化生产，发展高档蔬菜种苗和规模化生产。台安继续扩大鸭鹅饲养规模，发展蔬菜和花卉种植业。岫岩进一步夯实全国“蘑菇第一县”地位，大力发展草腐菌品种和工厂化栽培。坚持农业品牌化发展，全年打造5个省级名牌农产品，3个省级农产品特产之乡。

加速县域农高区建设。海城农高区重点推进三星公司上市，打造现代农业示范区和新型城镇先导区。台安农高区重点打造东北最大畜禽产品精深加工示范区。岫岩农高区重点打造全国最大食用菌及深加工产业示范区。全年县域开工建设亿元以上农业产业化项目30个，新培育规模以上企业20家。

加速农业生产经营方式转变。全面深化农村金融、农业保险体系改革。推进农村土地集约化经营，积极发展专业大户、家庭农场、农民专业合作社、农业企业等农业新型经营主体。巩固水稻全程机械化成果，努力实现粮食丰收，切实保障粮食安全。与省农科院、沈阳农业大学合作创建科技共建平台，加快农业新品种、新技术、新装备引进、示范、推广。

（五）全力推进新型城镇化

按照区域一体、产城融合、以人为本的发展思路，加强规划引领，统筹推进新型城镇化建设。

区域一体推进城镇化。明确新型城镇化城镇体系，推动中心城区、县城、新城新市镇、镇村合理分工、协同发展。以鞍海一体化为龙头，以新城新市镇建设为重点，加速铁东中央商务区、汤岗子中央活力区和县域行政中心、商务中心建设，带动工业园区、服务业集聚区同城化发展。以新一轮城市总体规划修编为契机，加强对城乡总体规划、资源利用规划等深度研究，科学规划新型城镇化建设布局，切实加强城乡规划管理，做到一张蓝图画到底。

产城融合推进城镇化。以汤岗子新城健康养老、文化旅游，达道湾、腾鳌新城钢铁深加工、精细化工和高端阀门，海西新城服装箱包，析木新城菱镁滑石，八角台新城农副产品物流集散，玉都新城宝玉石加工集散，牛庄新市镇古城旅游为重点，加速推进新型工业化、服务业集聚化与新型城镇化互动发展。

以人为本推进城镇化。坚持以人为核心，用产业发展吸引人口集聚，就近就地转移农民。加快基本公共服务体系建设，推进鞍海公共服务均等化，尽快实现农业转移人口市民化。做到以产兴业，以业聚人，以人兴城。全年力争县域房地产开发面积1000万平方米以上。

推进基础设施一体化。建立透明规范的城市建设投融资机制，完善中心城区基础设施，重点推进新城新市镇和开发园区基础设施建设，加快医院、学校等配套公共服务设施建设。加速建设兴业大道、建国大道、四达大道、鞍山路、劳动路、机场路、万水河南北路等61项续建工程。推进自由东街隧道、解放东路大修、解放路立交桥改造等81项新开工建设项目。推进地下管网改造。维修改造100公里村级公路，改造7座公路险桥。加快建设大伙房水源供水、汤岗子新城一期供水和燃气储配站，加速推进国电鞍山热电联产和中电国际汤岗子热电联产等公用基础设施建设。

（六）全力提高人民群众生活质量

以为城乡群众办20件实事为重点，加快民生工程和社会事业建设，满足人民过上美好生活的新期待。

全面实施民生工程。加大就业源建设，做好大学毕业生、新生劳动力和就业困难群体就业工作。加强技能培训和创业培训，以创业带动就业。全年实名制就业10万人，城镇登记失业率控制在3%以内，继续保持“零就业家庭”动态为零。扩大新型农村社会养老保险、城镇居民医疗保险大病统筹覆盖面，推进“五险合一”社会保障信息化建设。城乡最低生活保障标准提高10%以上。加强城市道路交通规划和管理。新增200辆清洁能源公交车，新开通、延伸6条市区公交线路，新建30个农村客运候车亭。支持少数民族地区发展，不断提高少数民族地区人民生活水平。发展社会养老事业，改造20所农村敬老院，提高高龄老人生活补贴标准。推进新殡仪管理服务中心建设，建成城乡公益性公墓。

加速改善生态环境。大力实施蓝天工程，完成鞍钢21座石灰竖窑拆除、3台烧结机脱硫改造等治理项目，加强监管，确保已建成项目正常运行，加大气化鞍山、扬尘污染防治力度，严格监测和控制城市PM2.5。深入实施青山工程，全年完成荒山荒地造林6.5万亩，退坡地造林1.7万亩，墓园绿化2万株，矿山生态环境治理3000亩，做好城市周边部分生产矿、闭坑矿植被恢复示范项目，启动10万亩海岫经济林产业带建设工程。加快实施碧水工程，新建运粮河永宁污水处理厂，完成城区6个污水处理厂截流干管建设，城市污水处理率达到95%以上，积极推进凤凰湖、万水河等水系公园和辽河台安生态文明示范区建设。开展农村环境整治先进村试点，对300个村屯进行综合整治。

加快发展社会事业。深入推进基础教育强县区创建工作，新建改造10所公办幼儿园，培训1万名农村教师，实施城乡学校互助计划。改善高中办学条件，推进普通高中多元化特色发展。深化职业教育改革，创建全省职教示范园区。支持辽宁科技大学发展和鞍山师范学院新校区建设。探索文化体制改革，鼓励非公有制经济发展新型文化产业，提高文化软实力。启动文化中心、规划展示中心建设，完成标准化乡镇中心文化站和电子阅览室建设。深化医药卫生体制改革，推进社会资本参与市中心医院、中医院、汤岗子疗养院等公立医院改革。推进鞍山医院、结核医院、精神康复医院建设，改扩建市妇儿医院。开展全市城乡65周岁以上老年人免费健康体检。完善全市急诊急救体系建设。积极备战省十二届运动会，维修更换城乡健身器材，广泛开展全民健身活动。

不断强化社会治理。大力推进平安鞍山建设，创新社会治理体制。以加强社区建设和管理服务为重点，探索建立政府治理和社会自我调节、居民良性互动的社会治理方式。推进“六五”普法，建设法治城市。加强市场监管，强化食品药品质量安全检查，加强病死动物无害化处理，严厉打击生产销售假冒伪劣食品药品行为。加强安全生产和交通消防安全隐患排查治理，坚决遏制重特大生产安全事故发生。进一步完善社会治安立体防控体系，依法严厉打击各类违法犯罪活动。充分发挥人民调解、行政调解、司法调解作用，强化社区矫正工作。加大信访问题化解力度，创造和谐稳定社会环境。

进一步做好广播电视、新闻出版、人口与计划生育、妇女儿童、老龄、残联、民族、宗教、外事、侨务、对台、统计、接待、史志、档案、气象、地震、人防、民兵预备役、双拥共建等工作。

（七）全力建设为民务实清廉政府

以开展党的群众路线教育实践活动为契机，加强政府自身建设，用实实在在的业绩，接受党和人民的检验。

建设法治政府。依法接受市人大及其常委会法律监督、工作监督和市政协民主监督。政府重大决策向人大及其常委会报告，向政协征求意见。办好人大代表议案、建议和政协提案。广泛听取各民主党派、工商联、无党派人士意见和建议。发挥法律顾问作用，建立健全政府涉法事项风险防范机制。完善行政执法程序，严格规范行政执法行为。

建设诚信政府。完善政府科学决策机制，对涉及公众利益的重大决策，实行事前论证、事中监督、事后评估，广泛开展听证论证会，自觉接受群众监督、舆论监督和社会监督。建立公开公正的信用环境，对政府出台的政策，对企业、群众和投资者作出的承诺，坚决落实到位，以政府诚信带动社会诚信。

建设责任政府。结合群众路线教育实践活动，认真解决“四风”问题。大力倡导勇于担当、敢啃硬骨头的精神，坚决做到不回避矛盾、不绕开问题、不推卸责任、不做表面文章。深刻认识事物发展客观规律，善于用改革思维和创新方法破解难题。

建设廉洁政府。进一步深化“五大系统”建设，全面开展制度廉洁性评估，推进网上审批和行政权力电子监察。在干部队伍中，集中开展治庸、治懒、治散、治浮、治奢专项整治行动，加大行政问责力度。全面开展职能部门自检自查，坚决杜绝“门难进、脸难看、事难办”现象，提高政府服务质量和行政效率，努力营造良好发展软环境。

各位代表、同志们，让我们以党的十八届三中全会精神为指引，在市委的正确领导下，凝心聚力，共谋发展，为全面建设“五个鞍山”，实现鞍山老工业基地全面振兴而努力奋斗！
